# Flourishing in Resonance: Joint Resilience Building Through Music and Motion

**DOI:** 10.3389/fpsyg.2021.666702

**Published:** 2021-05-31

**Authors:** Luc Nijs, Georgia Nicolaou

**Affiliations:** ^1^Institute of Psychoacoustics and Electronic Music (IPEM), Ghent, Belgium; ^2^Artesis Plantijn Hogeschool Antwerpen, Royal Conservatoire Antwerp, Antwerp, Belgium

**Keywords:** eudaimonia, resilience, children at risk, music and movement, embodied music cognition

## Abstract

Worldwide, children face adverse childhood experiences, being exposed to risks ranging from, exposure to political violence and forced migration over the deleterious effects of climate change, to unsafe cultural practices. As a consequence, children that seek refuge or migrate to European countries are extremely vulnerable, often struggling with integration in school, peer community, and their broader social circle. This multifaceted struggle can derive from external factors, such as the adaptation process and contact with other children, or internal factors such as the fears and trauma that every child carries within them since they departed from their homeland. To bounce, grow, connect, and create in both adversity and opportunity, children need to build resilience, i.e., the capacity of an individual to maintain stable psychological functioning throughout the course of adversity. On the one hand, building resilience requires developing a set of individual skills (internal protective factors), such as self-control, emotion regulation, self-esteem, and agency. On the other hand, building resilience involves developing social skills (external protective factors), connection, and close relationships. In this theoretical contribution, we review and map existing research to argue that activities based on the combination of music and movement has a strong potential to intensively build resilience. First, we connect the concepts of resilience and eudaimonia, based on the protective factors and key components of resilience. Then we discuss how music and movement, separately, may contribute to building resilience. Next, drawing on the basic mechanisms of musical sense-making, we argue that through combining music and movement, children engage in empowering musical sense-making processes that support building resilience, and in this way, support them to grow together and deeply experience eudaimonic values such as self-awareness, confidence and self-esteem, personal autonomy, connection, belonging, and bonding. Finally, we connect theory to practice. Based on the presented theoretical elaborations and on the authors’ experience as practitioners, we propose a set of guiding principles for the design of movement-based musical activities that foster the internal and external factors necessary to build resilience.

## Introduction

Worldwide, children face adverse childhood experiences, being exposed to risks ranging from political violence and forced migration over the deleterious effects of climate change, to unsafe cultural practices (Unesco, 2019). According to the European Commission (2020), 40% of the forcibly displaced population is children. Indeed, there is an intense international concern for the protection and well being of children and adolescents, since they are frequently among the most seriously impacted by these adverse conditions due to their youthfulness and lack of social power (Boyden and Mann, 2005).

Many children seek refuge or migrate to European countries, often arriving unaccompanied, traumatized by the violence they faced and the loss of their beloved ones. Once arrived in Europe, the struggle goes on, facing difficulties with integration in school (Crul et al., 2019), peer community (Daniel et al., 2020), and their broader social circle (Lundberg, 2020). For example in Greece, one of the countries that has been receiving hundreds of thousands of refugees over the past years, it is clearly evident that refugees remain socially excluded, despite the various humanitarian programs that have been developed to support people’s lives (Bareka et al., 2019). Refugee children usually suffer from social exclusion due to their families’ position in the society, besides the already present, escalating symptoms of post-traumatic stress disorder, depression, anxiety, somatic complaints, and behavioral problems (Ehntholt and Yule, 2006; Bareka et al., 2019). Moreover, the fact that the education systems are found unprepared to cope with the unprecedented numbers of refugee children, leads to abundant challenges in the education systems (Kostoulas-Makrakis and Makrakis, 2020). Education is not only a basic right, but a basic need for their social and emotional well-being of children, that strongly influences their academic outcomes and future integration in the society (Cerna, 2019). Childhood educators can play a vital role in assisting young refugee children impacted by war and conflict by understanding the consequence of this trauma on their psychosocial well-being (Murray, 2019). Through properly designed educational programs that support wellbeing and promote resilience, children can learn how to cope with the adversities they faced and they can start building their own future, making meaning in their current social context (Shallow and Whitington, 2014).

Building resilience is therefore of critical importance. It not only helps to cope with current difficulties as part of everyday life, but also to acquire the basic skills and habits that allow dealing with future challenges. Evidently, children do not start at zero after arrival in a host country. They bring along traditions, experiences, their language, beliefs and values, stories and songs, all being part of their cultural wealth (He et al., 2015; Kenny, 2018). This induces a renegotiation of cultural meanings and norms, which is arguably an important part of building resilience (Kenny, 2018; Erdemir, 2021).

As resilience is a complex phenomenon, there is not one way to build it but rather it’s development is the result of the strengthening of internal (individual) and external factors (family, community, and environment) (Barankin and Khanlou, 2007). Ungar and Theron (2020) mentions three approaches to help build resilience, by helping them to succeed in (1) navigating to the personal and social resources needed to experience well-being and (2) negotiating for resources to be provided in ways that are meaningful to children and youth from diverse contexts and cultures. The first, and most common, approach concerns *direct clinical practice*, focused on changing thoughts, feelings, and behaviors. The second approach concerns *case management*, focusing on helping people to develop the life skills and networks necessary to live independently. The third approach concerns *community work*, focusing on work with groups of people that experience a common challenge, or the wider community.

Here, we address the third approach as a way to work with children at risk, facing similar adversities, such as refugee children. We focus on a specific type of community work, namely making music together. Moreover, we address a specific aspect of music making, the role of bodily interaction in joint engagement with and through music.

The main point we wish to make in this article is that activities based on the combination of music and movement have a strong potential to intensively build resilience. The basic idea is that that if movement helps to build resilience, and music helps build resilience, then music and movement may have an intensified effect in supporting building resilience.

To make our point, we discursively elaborate on the empowering nature of interacting with music as a way to build resilience, thereby reviewing and mapping existing research. Starting from the connection between eudaimonia and resilience, we elaborate on how music and movement contribute to this eudaimonic perspective on building resilience. Next, we describe the empowering nature of musical interaction and it’s bodily basis by elaborating the mechanisms and processes that underlie meaningful engagement with music. Finally, we propose a set of guiding principles to shape a movement-based approach to joint music making as a powerful way to build resilience.

## Eudaimonia and Resilience

To understand how music and movement may contribute to building resilience, it is useful to first connect the concept of resilience to the concept of Eudaimonia.

Facing difficulties in life, it is not easy to “function well” or to “actualize one’s potential,” both characterizing the concept of *eudaimonia* (Huta and Waterman, 2013). According to Ryff (2014), eudaimonia is related to processes such as meaning-making, self realization and growth, to quality connections to others, and to self-knowledge. These qualities may be particularly important when faced with challenges and difficulties in life. Indeed, as different scholars assert, “functioning well” also embodies flourishing in difficult times [see Cruywagen (2018)].

As such, eudaimonia and resilience are closely interrelated (Cho and Docherty, 2020; Sirgy, 2020). On the one hand, resilience reflects the ability to retain, restore, and balance one’s level of well-being when a deviation occurs due to adverse circumstances (Cummins and Wooden, 2014). On the other hand, eudaimonia can be considered a mental resource, involving a set of traits that provide positive resources to cope with such circumstances, but also to promote the mitigation of future stress (Masten, 2007).

To overcome obstacles in life and construct a healthy environment that fosters prosperity and well-being, resilience, i.e., an individual’s capacity to maintain stable psychological functioning throughout the course of adversity, is necessary (Marley and Mauki, 2018). Resilience refers to positive outcomes and transformation, or the realization of specific landmarks of the development of an individual having encountered major risks, hardships, and stressful situations (Naglieri et al., 2013). It can be conceived as an enduring characteristic of an individual, a circumstantial interaction between the individual and its environment or a unitary establishment which can be adequate for several settings, such as social, as well as academic (Condly, 2006).

Since risks are multifaceted, resilience cannot be conceived to be single-sided. It is complex and multidimensional, altered by personal, material, social, and cultural assets among significant loss (Ungar, 2006; Ryan et al., 2008; Robertson and Cooper, 2013; Lenette et al., 2015). It is contextual, situated in everyday personal practices and therefore considered vital in the process of adaptation to change and in dealing with adversity (Wagnild and Collins, 2009; Lenette et al., 2013).

### Protective Factors

Resilience is the result of a combination of protective factors, operating at different levels and leading to positive outcomes (Marley and Mauki, 2018). According to these authors, focusing on resilience and its protective factors is therefore an essential long-term objective for establishing a positive mental health and psychological functioning. It is the combination of resilience, risk factors (e.g., overty, violence, broken families, and discrimination) and prospective factors that determines how flourishing life can be. Risk factors concern short-term or long-term threats to individuals’ well-being, increasing the probability of negative outcomes. Protective factors can function as a buffer to risk factors, interrupt their cumulative effects or intervene to prevent a risk factor from having a negative effect on the development of the individual (Barnová and Tamášová, 2018).

The protective factors can be broadly categorized into internal and external factors. Internal factors concern, for example, self-control, emotion regulation, motivation to succeed, and self-efficacy (Weir, 2017). Also self-esteem is an important protective factor (e.g., Kidd and Shahar, 2008; Balgiu, 2017; Arslan, 2019). High self-esteem contributes to positive emotion (e.g., Goodall, 2015), positive relationships with others (Harris and Orth, 2019), self-determination and successful coping with threats (Leary and Baumeister, 2000), and higher academic achievements (Cvencek et al., 2018). Also agency has a considerable impact on positive emotion and well-being. For example, Veronese et al. (2017) show that the agency among Palestinian children plays an important role on their emotional well-being and personal development. Karlsen and Westerlund (2010) also explain how joint musical interaction promotes agency on both the musical and social level.

External factors concern social skills, connection, and close relationships (Marley and Mauki, 2018). The factors that influence resilience are often related to the attachment system, for example having supportive parents or primary caregivers or having close relationships with other caring adults and close peer relationships (Garmezy, 1987; Riley and Masten, 2005). During childhood, finding comfort in secure attachments is essential. The availability of a close, compassionate, regularly attentive, and nurturing parent or caregiver is most valuable to the sense of confidence and self-esteem of an individual or a child (Erikson, 1993; Levine, 2003). For example, factors such as family cohesion, residence stability and the integration of families into host communities can positively influnce social-emotional well-being in newly arrived refugee children (Zwi et al., 2018).

Equally important is the impact of factors that extend beyond the family, such as influential schools and neighborhoods and the elements of faith and hope embedded in spiritual and cultural beliefs (Weir, 2017). The latter is important, as children may live simultaneously in multiple communities: the community they live in, the cultural community they identify with, their community of peers, or a community formed by exclusion (e.g., when grouping together due to being marginalized by ability or sexual orientation). Note that the children, who find themselves often in two or more communities synchronously, may encounter difficulties in identifying the values that are relevant in the process of their own positive growth. Yet, such profusion may generate more pathways to resilience (Ungar, 2007). Indeed, we can assume that even socially unpopular conduct by a child or family that refuses intervention or puts the child further in an unsafe situation, can be a secret road to resilience (Dyer and McGuinness, 1996; Ungar, 2006; Stieglitz, 2010; Capous-Desyllas and Barron, 2017). Additionally, extensive research exemplifies that exposure to risk factors can influence positively the resiliency of children as well as their adaptation to a variety of stressors such as poverty, migration, and war (Garmezy, 1985, 1987, 1993; Masten and Garmezy, 1985).

Importantly, the protective factors appear to merge with one another according to age, stages in life and influences of every child, thus the origin is more complex and therefore cannot be clearly categorized (Condly, 2006). Moreover, neither the internal nor external factors alone are likely to ensure positive outcomes for children who experience prolonged periods of adversity. It is the interaction between biology and environment that builds a child’s ability to cope with adversity and overcome threats to healthy development. For instance, in the case of refugee children, one should consider both the individual characteristics as well as the wider community factors and beliefs, alongside the level of exposure to traumatic events (Ehntholt and Yule, 2006).

Nevertheless, according to Masten (2001), the most widely reported predictors of resilience strive to be caring relationships, for example with prosocial adults, as well as satisfactory intellectual functioning (Masten and Coatsworth, 1998; Masten, 2001). For example, Rip et al. (2020) show that caring relationships with the foster families among unaccompanied refugee youth appear to be crucial for their adaptation and growth within their new environment.

### Key Components

Key components of resilience include *meaning making*, *self-realization*, and *growth*. In the process of becoming resilient, finding or creating meaning in life by confronting significant challenges is essential. For example, in line with Liebenberg and Joubert (2019) who argue that meaning making processes form an essential mechanism in the enactment of agency, shape how people make sense of daily life and support managing the adversities they encounter(ed), Goodman (2004) showed that meaning making is an important cooping strategy for unaccompanied refugee youths.

Equally important is creating *purpose in life*, which is the extent to which an individual recognizes the meaning, purpose and direction in life (Ryff, 2013). This relates to self-realization, i.e., the ability to creatively and positively pursue development, psychological maturity and competence, while re-establishing the power of individuality (Maksimenko and Serdiuk, 2016), and personal growth, i.e., the extent to which an individual utilizes his talents and full potential (Ryff, 2014). These processes involve elements such as self-determination, self-organization and competence. Such aspects for instance, are essential for enhancing the psychological well-being of children who live in detention like refugee camps (Veronese et al., 2020).

Empowerment, the process of transformation that allows a person to make autonomous and responsible decisions, appears to be another key component of resilience. Indeed, having control over the self leads ultimately to autonomy which involves the experience of integration and freedom that is an essential aspect of healthy human functioning (Deci and Ryan, 2000). Core mechanisms of empowerment are agency, self-knowledge, and the presence of an environment that encourages change (Hennink et al., 2012). Moreover, the occurrence of empowerment at an individual level facilitates empowerment at the level of the group and/or the community and vice versa, i.e., the person and the social system or community in which a person lives are symbiotically connected (Cadell et al., 2009). Therefore, its impact can extend to the broader social circle and the society.

Other components of resilience encompass bonding, sense of contribution and belonging, hope and meaningfulness in life (Huta and Waterman, 2013; Ryff, 2013). Together, these components contribute to the protective mechanisms that counter risk factors (Lenette et al., 2015). These mechanisms have also been identified as unique competences that are required for the resilience process to take place (Dyer and McGuinness, 1996).

### Building Resilience

Several studies have demonstrated how resilience is built by strengthening the above described protective factors and key components through intervention-based studies, often based on specific educational programs. For example, the educational programs developed by *Right to Play*, an international non-governmental organization that aims to educate and inspire children to lead happier, more empowered and safer lives by employing the motivational force of play and sports^1^. Their programs have been introduced in several school settings as well as groups of refugee children and children in war zones, and proved to have a positive impact on specific factors of resilience. For example, a study using a Right To Play program with Syrian refugee children in Lebanon, revealed that play has an overarching impact on resilience, facilitating positive psychosocial results in developmental pathways, including trauma rehabilitation, literacy, compassionate action, social justice, and prosocial competitiveness among children (Cook, 2017). In addition, over time, a beneficial effect of play on protective factors could be maintained, indicating that it could serve as promotive factors at the individual level, with sustained coping skills and an improved capacity to thrive in challenging circumstances (Cook, 2015, 2017; Yaylaci, 2018).

Next to play, also the arts can contribute to building resilience. Indeed, a systematic research by Marsh (2006, 2008, 2012, 2017) outlines that for many refugee and newly arrived immigrant children in the process of relocation, music, movement, dance, and play, as vital forms of human experience, offer essential ways to communicate with others, gain comfort, convey emotions and develop self-esteem, identity, and resilience. Creative participation in music and dance can give highly vulnerable refugee children a degree of agency, allowing them to mediate negative effects of forced displacement in ways that are meaningful and of their own choice.

To conclude, Masten (2001) exquisitely defines that resilience comes “from the everyday magic of the ordinary,” such as the regulating human resources of the children, the way they think, function and act and the way they interact within their families, relationships and their communities. Thus, the priority should be to foster adaptive systems where positive feelings can emerge, taking into account the environmental and developmental context of children. In this way, growing resilient children can thrive and flourish into a prosperous future, being spiritually mature, self-determined and experiencing quality connections with others. In the next sections, we elaborate on the strong potential of music and movement to contribute to building resilience and promote eudaimonia.

## Music and Resilience

The connection between resilience and Eudaimonia, is a good starting point to consider the potential of music to support building resilience.

Looking back at the New Deal Era until the end of World War II, American scholars Willem van de Wall and Max Kaplan viewed music as an essential ingredient to the health and well-being of a society (Krikun, 2010). Indeed, an increasing number of research studies shows how engaging with music contributes to a flourishing life. Already mere listening to music leads to some happiness, contributing to pleasure, engagement, and meaning (Seligman, 2004; Lamont, 2011). The accompanying associations (e.g., to important moments in life), chills or state of bliss may—in the short run—translate into momentary feelings of *pleasure*, but—in the long run—also imbue daily life with meaning and purpose and lead to eudaimonia (Stark et al., 2018). Arguably, music can indeed bring some magic to the ordinary.

At the fundamental level, the power of music to promote eudaimonia is related to the “sweet anticipation” of the unfolding music, i.e., “the positive feelings that arise from conscious thought about some future event” (Huron, 2006). This aspect is further elaborated on in the section “Music, Movement, and Resilience.” Focused self-chosen music listening also promotes *engagement*, through eliciting flow experience (Csikszentmihalyi, 2002). Furthermore, music may contribute to *meaning* by, for example, shaping important moments in life (e.g., rituals), promoting a sense identity, and as such by stimulating personal growth (Lamont, 2011). Finally, listening also contributes to *social relatedness*, enabling to feel close to friends, to express one’s identity and values, and to get familiar with their social environment (Schäfer et al., 2013). For example, Millar and Warwick (2019) emphasize the significant function of music in the development of supportive social environments within the context of refugee camps.

Although passive engagement with music likely triggers the same neural pathways involved in active musical interaction through the activation of motor regions of the brain during music listening (Levitin and Menon, 2003), active music making may contribute even more to progress on the pathway to eudaimonia through nurturing resilience. Despite a growing number of studies, in comparison to music listening and music therapy, less is known about the effects of active music making on well-being in everyday life or its underlying mechanisms (Koehler and Neubauer, 2020). However, it might be argued that active music making not only may reinforce or intensify aspects of music listening (e.g., prediction, anticipation, engagement, bonding, and positive emotion) but also may contribute to a sense of achievement or accomplishment. Croom (2015) convincingly offers support for the claim that music practice and participation can positively contribute to living a flourishing life by positively influencing emotions, engagement, relationships, meaning, and accomplishment. Especially the latter, accomplishment, plays an important role as it introduces agency in relation to the other aspects of eudaimonia (Biesta, 2008). For example, Kenny (2016) describes the spaces in asylum centers where music making takes place as “communities of musical practice,” where children have the opportunity to gain agency and control through musical choices. Additionally, Karlsen (2013) highlights that such pedagogical frameworks should promote diversity by responding to the students’ choices and encouraging experimentation through shared musical experiences. Similarly, the idea of a “community of musical practice” appears in the form of a “cultural and performative space” emerging from accidental communities in detention in the study by Weston and Lenette (2016). The asylum seeking participants share musical experiences which lead to finding a common ground in the most unnatural space, allowing democratic expression and facilitating a sense of well-being (Weston and Lenette, 2016).

Considering the above, active engagement with music, particularly in group settings, assumedly contributes to both internal and external protective factors of resilience. With regard to the internal factors, active music playing has been linked—among others—to self-control, self-esteem, and self-efficacy [see also Daykin et al. (2011) and Hallam and Council (2015)]. For example, Hietolahti-Ansten and Kalliopuska (1990) observed that expressing oneself through music promotes responsibility, and concentration, and improves inner self-control. Also Rickard et al. (2013) and Zapata and Hargreaves (2018) found that musical activities had a significant impact on children’s self-esteem. Yun and Kim (2013) found that children’s self-expression, self-efficacy, and social skills increase after musical activities based on the Orff approach. Ritchie and Williamon’s study on self-efficacy showed that children who are involved in musical activities report higher self-efficacy than those who aren’t (Ritchie and Williamon, 2011). According to Kenny (2018), collective music making among refugee and asylum seeking children can enhance a sense of identity, agency, belonging and togetherness while leading to life quality improvement within the limits of their temporary living arrangements (Kenny, 2018).

With regard to the external factors, music playing has been linked to the development of social skills and prosocial behavior. For example, Schellenberg et al. (2015) showed that music training in a group facilitates the development of prosocial skills. Also Beck and Rieser (2020) provided evidence that music making may facilitate prosocial behavior in preschoolers. Furthermore, joint active music making fosters connection and close relationships, not only within the smaller community (e.g., family), but also in the broader community and even with strangers. For example, Boer and Abubakar (2014) found family and peer cohesion to be consistently and strongly related to the presence of music rituals within families and peers in Kenya, the Philippines, New Zealand, and Germany. Weinstein et al. (2016) showed that singing in a choir promotes, next to positive affect, feelings of inclusion and connectivity. Howell’s multiple case study on community music at the Melbourne English Language School for newly arrived refugee and immigrant children, showed that playing and composing in a collective setting fosters connectedness (Howell, 2011). It further revealed that music has an enormous capacity to help these children build the self-esteem and resilience needed in coping with challenges in their education and life transition. Also Mosley et al. (2019) showed that long periods of exposure to community music lead to high aspiration levels, which are significantly associated with the development of resilience and social qualities of extroversion and the capacity for conflict resolution.

Despite the rich amount of studies and initiatives that seem to acknowledge the valuable potential of engagement with music to build resilience, we cannot but observe that an aspect that is underexposed in the literature is the role of the body in musical interaction, when using music to build resilience. This is rather remarkable, considering (1) the essential role of the body and body movement in musical interaction and the empowering nature of addressing this role, and (2) the growing body of research that shows how movement is related to resilience and to several of its components such as bonding or pro-social behavior. Therefore, in the next sections, we first elaborate on the connection between movement and resilience and then on the synergy between music, movement and resilience.

## Movement and Resilience

Humans are natural born movers and our body plays a fundamental role in the dynamic interaction with the world (Johnson, 2007). In essence, the body is our vehicle of being part of and experiencing the world (Behnke, 1989; Dant, 1999; Dant and Wheaton, 2007). Next to being vital to surviving and functioning in a complex environment, our body and body movement provide the primary sphere in which all meaning is initially engendered (Merleau-Ponty, 1945). Moreover, the way our body learns to be in the world connects to our desire to engage in interacting with it and the pleasure derived from that interaction (Dant and Wheaton, 2007).

Given the important role of the body and body movement in our being in the world, it is almost evident to say that the bodily activities may contribute to well-being through physical health. However, the role of the body and, in particular, body movement in establishing well-being goes beyond physical well-being. Indeed, there is a body of research that indicates the potential of engaging in movement activities to promote mental, i.e., emotional, social and psychological, well-being. For example, dancing is regarded as a multidimensional practice that contributes positively to many facets of human well-being (Quiroga Murcia et al., 2010). A systematic overview by Sheppard and Broughton (2020) shows how studies reveal the positive impact of dance on, for example, quality of life or sense of life satisfaction, on social engagement and building communities, and on body image and self-acceptance. Also physical activity/sports prove to have a positive impact on well-being [see systematic reviews by Eime et al. (2013), Lubans et al. (2012), and Zhang and Chen (2019)] and has even been recognized as one of the most influential factors for improving psychological well-being (Zhang and Chen, 2019). Furthermore, van der Kolk’s work on trauma and the role of body in the healing process portrays how holistic body practices such as yoga can significantly improve PTSD (post-traumatic stress disorder) symptoms, enhancing self-regualtion and self-awareness through promoting a deeper connection with the body and mind (van der Kolk, 2014, pp. 214–220).

Viewing the intrinsic link between the body, cognition and emotion, and taking into account the positive impact of physical/movement activities on well-being, the idea that movement is a powerful form of expression can be closely linked with enhancing internal and external factors of resilience. With regard to the internal factors, Feldman (2020) showed that body ownership and agency increase while moving spontaneously and authentically within a group. Theodorakou and Zervas (2003) investigated the effect of creative movement in physical education on self-esteem. They concluded that movement activities positively influence the cognitive, social and physical aspects of self-esteem, with more effect when creative movement is used. This resonates with Feldman, who shows that creative play during movement interventions fostered connection, spontaneity, and choice-making in the present (Feldman, 2017). Moreover, there is considerable evidence that movement synchrony can enhance internal factors of resilience. For example, Launay et al. (2013) suggest that synchronization can enhance feelings of trust and agency. Similarly, van der Kolk (2014) shows that sychronicity in movement during physical theatre activities can promote feelings of trust and safety among traumatized youth. Stark et al. (2018) suggest that exertion and synchronization have a positive effect on endorphin release which is connected to the sense of “feeling good.” This aspect is also evident in the study by Tarr et al. (2015) who suggest that exertion and dancing in synchrony can enhance endorphin release and have beneficial independent effects on social bonding.

Synchrony in movement has also a beneficial impact on external aspects of resilience too. Tunçgenç and Cohen (2016) found that active synchrony in groups of children promotes spontaneous helping and social bonding. According to Valdesolo and DeSteno (2011), synchrony can differentially engage socially oriented emotions such as compassion and liking, thus influencing prosocial and cooperative behavior. Beck (2018) also provides evidence on the correlation between synchrony and prosocial behavior in joint movement activity with preschool-aged children. The work of Rabinowitch and Meltzoff (2017a, b) further demonstrates the effect of synchrony in cooperative behavior in preschool-aged children. For example in their studies with 4-year children they show that joint synchronized movement assisted in completing the tasks faster, indicating peer cooperation, intentional communication, and sharing (Rabinowitch and Meltzoff, 2017a,b). In addition, a study by Vacharkulksemsuk and Fredrickson (2012) with strangers randomly assigned in dyads to carry out tasks, reveals that behavioral synchrony acts as an autonomous method to improve the quality of social interactions, by promoting positivity, mutuality, and vitality in connections.

A strong body of literature about resilience through movement or dance, focuses on the implementations of dance movement therapy (DMT) through shared movement experiences and its therapeutic quality (Samaritter, 2019). DMT is a dynamic form of therapy that works principally with the non-verbal, movement-based expression of the patient and the movement and verbal input of the therapist. The DMT interventions aim to guide the patient through a positive developmental process, or they assist in working through trauma, often being accompanied by introspective insight-focused counseling (Wengrower, 2009). Several studies focus on how the DMT interventions can promote internal factors of resilience such as self-control, emotion regulation, problem-solving (Koshland, 2010), self-esteem (Wengrower, 2015), and empowerment (Levine et al., 2015).

For instance, in the recently conducted study by van Geest et al. (2021) the interbodily feedback between the dance movement therapist and the participant proved to enhance happiness and therefore well-being. Additionally, Verreault, in her study with female asylum seekers and refugees in Netherlands, found that emphasizing the cultural contexts, imagination and resources of the participants through bodily interaction during the DMT sessions could relieve tension, overcome vulnerabilities, improve self-agency, help to restore a sense of body autonomy and thereby promote resilience (Verreault, 2017). Besides the promotion of the above mentioned internal factors of resilience, the external factors were also evident in the sense of togetherness and belonging while moving together in a group. The study further revealed that the non-verbal nature and movement-based focus of the approach can complement a resilience oriented framework, providing group support and creating a safe environment for cross-cultural groups and populations where anguish is manifested somatically.

Next to DMT, community dance projects have proved to empower people giving them a sense of contribution and belonging to a group. Monteiro and Wall (2011) support that traditional African dance can transform and empower the individual and subsequently the group. Houston further suggests that community dance has not only the capacity to transform and empower people, but also to enhance confidence (Houston, 2005). Important to mention are some studies looking into the use of a particular style of community dance, *Capoeira*, as a creative activity in engaging youth for building resilience. Capoeira is a traditional Afro-Brazilian combat dance often combined with playing music and singing (Assunção, 2005). For example the study of Momartin et al. (2018) shows that the healthy group environment fostered by the Capoeira practice taught young refugees to be resilient and respect each other, through promoting positive examples of subsistence and utilitarian relationships. Besides, Momartin et al. (2018) argued that due to Capoera’s unique framework of empowerment and confidence building, young refugees could overcome adversity through developing individual inner strength and group membership. Additionally, the study of Hast (2019) with the Za’atari refugee camp children, indicated that the embodied experiences of Capoeira create a sense of belonging, community and a new family.

## Music, Movement, and Resilience

The ability of music on the one hand, and movement on the other hand, to support building resilience and as such to foster a flourishing life, invites us to further consider the intrinsic relation between music and movement as a powerful mechanism that supports and promotes building resilience. In recent years, this intrinsic relation has gained much interest in the domain of music research (e.g., Gritten and King, 2006; Lesaffre et al., 2017). In the domain of music education, the tight relationship between music and music, and the potential of movement to develop musical skills has been acknowledged for long time. Maybe the most elaborated approach, is the Dalcroze approach in which “movement is simultaneously a means of personal, social, and musical discovery” (Juntunen, 2016, p. 143). Interestingly, the Dalcroze approach has been found to have a beneficial impact on children’s wellbeing, bringing for example a sense of freedom and contentment (Habron, 2014). Indeed, according to Dalcroze himself, the way to health was through a balance of mind, body, and senses, and this can be achieved through music and movement activities (Altenmüller and Scholz, 2016).

In this section we elaborate on the power of music through expressive alignment, as elaborated by Leman (2016).

### Empowerment Through Music

Interaction with music is an empowering activity, emerging from a meaningful engagement with the music. According to Leman (2016), the empowering nature of our interaction with music stems from a specific state of equilibrium, or: homeostasis, in which our bodily engagement with the music plays an important role. Such a state is established through a cognitive-motivational loop. The cognitive aspect relates to the regulation of pattern processing in the expressive interaction with the music. The motivational aspect relates the regulation of arousal, valence, and engagement in relation to this pattern processing. Importantly, the driving and regulating force of this loop is expression. During such equilibrium state of expressive interaction, predictive, energetic and affective states intertwine, reinforce each other and, as such, generate a powerful rewarding condition that leads to empowerment (Leman, 2016).

The cognitive motivational loop is established throughout the expressive alignment with the music, which is based on the process of enactment (Leman, 2016). Enactment generates meaning in music.

### Musical Sense-Making

The basic idea is that, while interacting with music through listening, dancing or playing, we make sense of the music and attribute meaning to it through the establishment of a sound-movement-intention connection. This connection allows transforming the stream of sounds into a meaningful musical experience, through the association of sound patterns (e.g., chord sequence or melody) with movement patterns (e.g., shape, direction, energy) and thereby with the intentional states (e.g., an emotion) that underlie these patterns (enactment process). This connection is enabled through specific features that music and movement share (Sievers et al., 2013). For example, both modalities are time-based and thus music can be experienced as a flow of movement, imbued with a certain quality and with an intentional direction that can evoke an emotion (Stern, 2010). As such, experiencing the music through the body by exploring music’s connection to time, space, and physical energy is a powerful way to engage in musical sense-making (Jaques-Dalcroze, 1967).

### Basic Mechanisms

Enactment, or the process of attributing intentions to music by associating musical and movement patterns, is rooted in several basic mechanisms (Leman, 2016). A first basic mechanism, *prediction*, concerns the ability to sense what comes next in the music (e.g., how a chord progression unfolds) and the predict the outcome of a movement (e.g., reach a point in space in response to the beat). Within an embodied perspective, the prediction of how music unfolds is viewed as the expected outcome of *bodily-mediated* perceptions and physical actions with music, rather than the expected outcome of a direct line between music and the brain. Prediction is indeed embedded in sensorimotor schemes that establish tight couplings between motor commands and expectations (Pezzulo, 2011). These sensorimotor schemes originate from a repertoire of acquired actions (e.g., through deliberate practicing) and innate reflexes (e.g., postural or stretch reflexes). In this way, they involve kinaesthetic, tactile, and haptic sensing particularities of the body related to the biomechanics of the body (e.g., the length and form of our legs and arms; e.g., Dahl et al., 2014) and to the bodily states (e.g., feeling fatigued or being energetic) that drive the alignment. It is assumed that these particularities have an impact on the predictive processes and as such influence anticipation of expected outcomes (Clarke, 2005).

A second basic mechanism, *entrainment*, concerns the establishment of a global timing framework that emerges throughout the synchronization of movements with salient time markers in the music, such as the beat. Entrainment, or “the coordination of temporally structured events through interaction” (Clayton et al., 2004), involves being pulled toward synchronization, and as such helps the expressive alignment to music. According to Leman (2016), it enables three sensorimotor mechanisms: finding, keeping, and being the beat. Finding the beat is the process of recognizing the regularity in time of salient markers that allows keeping the beat, and eventually being the beat. Note that from finding to being, a transition occurs in effort. Finding the beat requires effort, but once the beat has been found and prediction runs smooth, it no longer requires effort, and energy is freed up to spend on other aspects of the musical interaction, for example the interaction with others. The attraction dynamics of entrainment are determined by natural motor variability (shaped by prediction and adaptation processes), motor resonance and preferred tempo (determined by biomechanics of the body and neuronal clocks) and body movement (e.g., by guiding attention) (Leman, 2016).

A third basic mechanism of the enactment process, *alignment*, concerns matching one’s physical actions to the music. Alignment can be viewed from the perspective of pattern matching, whereby movement patterns are corresponding in synchronization with salient time markers in music (*phase alignment*) or with what happens in-between the salient markers (*inter-phase alignment*) such as melodic, rhythmical, dynamical or harmonic structures. Next to the observable movement patterns, alignment also involves bodily states related to, for example, effort and arousal and known through proprioceptive observation (Leman, 2016). As such, it can be viewed from the perspective of states as embodied in patterns, and more specifically of state changes. These changes come about based on transition processes that contribute to the experience of music as a pleasurable and empowering phenomenon.

First, the ability to successfully align one’s movements to music induces a transition into feeling of being in control and to a sense of agency, causing feelings of satisfaction, reward and immersion (Clarke, 2014). This transition process is based on *predictive* processing.

Second, the physical effort required to carry out and maintain alignment can lead an increase in one’s arousal level. Physical activities to music may induce physiological and psychological states of being awake, alert and excited and, thereby, improve executive functions (Byun et al., 2014) and facilitate higher cognitive functions (Audiffren and André, 2015). This transition process is based on *energetic* processing.

Finally, music can affect the energetic state of a person by generating a transfer from sound energy to motor energy, based on the music’s qualitative features such as degree of variation, bass drum decibel level, length and structure of motives or timbre. This leads to the attribution of affect value to music, such as pleasant vs. unpleasant, happy vs. sad (Roda et al., 2014) and to a pro-social attitude. This transition process concerns *expressive* processing.

### Embodied Interaction for Eudaimonia and Resilience

Within the context of eudaimonia and resilience, the above described embodied nature of expressive interaction with music, involving the basic mechanisms of enactment and the transition processes that are involved in the expressive alignment to the music, are of particular interest, especially due to the coupling between bodily mechanisms or behaviors and music. Already present in listening but even more in playing music together, expression, physical effort, and control are intensely addressed and, as such, provoke deep feelings of pleasure, social connection, arousal, and power (Leman, 2016).

First, the rewarding nature of interacting with music plays an important role [see also Dubeé and Le Bel (2003) and Zatorre and Salimpoor (2013)], contributing to positive emotion (Geschwind et al., 2010; Shiota et al., 2014). Positive emotions are not only a core element of eudaimonia (Seligman, 2002) but also an important characteristic of resilience, enabling to rebound from, and find positive meaning in, stressful encounters (Tugade and Fredrickson, 2004). According to Leman (2016), this rewarding nature stems from the expressive alignment with music, based on the use of musical and movement patterns to enact musical expression. Feelings of reward through music are intrinsically related to the ability to anticipate and predict how the music unfolds (Huron, 2006; Salimpoor et al., 2015). As found in study van der Merwe’ (2015) on students first experiences with Dalcroze inspired activities, engaging in music and movement yields joyful experiences. Such positive experiences have a positive impact on, for example, “motivation, flexible learning strategies and self-regulation, and the availability of cognitive resources for task engagement” (Pekrun et al., 2009, p. 119).

Second, the basic mechanisms and transition processes involved in the expressive alignment of body movement to music, contribute to fundamental aspects of both internal and external protective factors. For example, the transition from pattern prediction into agency contributes to internal factors such as self-control, self-efficacy, and self-esteem. Indeed, a sense of agency involves controlling one’s actions and successfully influencing one’s functioning and the environment and it fosters self-efficacy, i.e., the belief in one’s ability to succeed in specific situations and to achieve one’s goals (Zulkosky, 2009; Bandura, 2010; Duggins, 2011). Furthermore, a loss of sense of agency may be accompanied with a loss of self-esteem (Unrau et al., 2008).

As the rewarding nature of musical interaction is, according to Leman (2016), related to the human—innate—expressive system, interacting with music appeals to the human urge to evoke expressive responses from others in order to establish an interaction that is rewarding for those who participate in the interaction. In this way, alignment to music contributes to the external factors of resilience, enabling the development of social skills, prosocial behavior and thus close relationships and connections. In particular, the reinforcement of expression through joint bodily interaction with music and the prosocial nature of the human expressive system in relation to the emotional palette of human interaction patterns, encourage others to participate in an interaction and provokes positive valence and arousal. In joint bodily interaction with music, individual sense of agency (“I did it!”) becomes valued within the collective agency of the group (“We did it!”) (Pacherie, 2014). According to McNeill (1995) such we-agency involves a euphoric feeling provoked by prolonged and rhythmic joint movement in a group that together stays in time. In this way the pro-social value of musical interaction is activated modulates the rewards that are associated with other interactive components, particularly the cognitive control (leading to agency) and the physical effort (leading to arousal) (Leman, 2016).

## Implications for Music Practices to Build Resilience

Acknowledging the above-described embodied nature of empowerment through music, it can be argued that combining music and movement in creative activities could intensify the process of building resilience.

Integrating musical activities in the daily lives of children, whereby the basic mechanisms and the transition processes involved in the enactment process are addressed through expressive alignment to the music, may boost the rewarding nature of children’s musical experiences, reinforce the development of the internal and external protective factors of resilience and bring some magic in the ordinary.

To address and implement the empowering nature of an embodied engagement with music, it is necessary to carefully consider how musical activities can be shaped so as to optimize the conditions for the development of the internal and external protective factors. In the following paragraphs we describe a set of guiding principles for the design of such musical activities. Aiming at supporting children at risk, such as refugee children, to build resilience, the presented principles are based on the evidence-based theoretical elaborations in the previous sections and aim at creating the conditions in which the protective factors can optimally be addressed in order to develop the key components of resilience. These principles are based on the studies and theories reviewed in the previous sections, and grounded in the pedagogical content knowledge of both authors. To illustrate the principles, Boxes 1–5 present possible group activities, the design of which is based on the description of the principle and inspired by the practical experience of both authors.

**BOX 1. Example activity to support connection.**

**Connect:** The children are asked to bring or name a recording of one or more traditional/folk songs or dances that represent their culture, it can be music their grandparents or parents play at home or sing with them. Excerpts of all the recordings are played by the facilitator and the children are asked to move freely in the room, aligning their movements to the music. When the music stops, they freeze, holding the position of that moment as if they are a statue. Then, they are asked to look around and take on the position of someone else. When the music starts again, they continue moving in the space. This is repeated several times and with different excerpts.

**BOX 1. Example activity to support exploration and experimentation.**

**Explore and Experiment:** The children are asked to identify one character - it can be a human or an imaginary creature - inspired from the music that has been just played and to start imitating it, through moving in space and producing sounds with their voice or by creatively using their body. They are then encouraged to choose one ‘prop’ from a collection offered to the whole group, including small (pitched) percussion instruments and other objects that can create sounds, colored pieces of fabric, and white paper with color markers. In this activity, they are completely free to express their ideas on creating this character through moving, creating sound, writing and drawing.

**BOX 3. Example activity to support blending.**

**Blend:** The children are randomly assigned to groups of three. To do so, they are invited to walk around in the room, while some music plays. When the music stops, they form a group with the two children that are nearest to them. They are then asked to share the material they created while exploring and experimenting and they are encouraged to expand it by introducing new musical ideas and by merging their existing material. Then, they are given a task to connect it in a mini-performance and present it to the rest of the group. After the presentation, a discussion follows, where the groups exchange opinions and ideas for further exploration.

**BOX 4. Example activity to support cooperation.**

**Cooperate:** The whole group of children is taken for a “soundwalk” outside. They are occasionally asked to stop, close their eyes and grasp their favourite sounds. They can then take time to record them with a tablet in duos. Then they are taken indoors and in a group discussion they are requested to think of an imaginary land or culture. They are encouraged to propose ideas on how they could use their inspiration from the “natural sounds” to create the landmark music and dance of this land, tribe or culture. They are free to make new, somewhat larger groups and form a new creation under one rule: the body has to be involved in the sound making (i.e. singing, speaking, stepping, and playing an instrument). They are further asked to create a graphic score that can reflect their composition in the best possible way.

**BOX 5. Example activity to support sharing.**

**Share:** The graphic scores (see Box 4) are exchanged between groups and every group has to interpret a new score using their voice, body and the small instruments to create sound. Every group records another group with a tablet. The videos and the graphic scores are then uploaded in a platform where all children have access to. They are also encouraged to comment and share ideas for further improvement towards a bigger live interdisciplinary performance where this material will also be incorporated. The parents/caregivers have also access to this platform and they can observe the process towards the final performance where they will have the chance to be present.

### Connect

A first guiding principle concerns the *connection* to the children that are invited to engage in movement based musical activities. This involves two main ways of connecting. The first way is to connect to the children’s social and cultural context. Indeed, every setting in which musical activities can be organized for children is different and children bring with them a series of embodied experiences related to their community with its sociocultural particularities. These particularities play an important role in the children’s expressive repertoire, as expressed in that qualities of gestures, actions and behaviors that align with the norms and values of the children’s communities. Note that this requires an assessment of not only the cultural norms, shared values, customs and beliefs of the specific environment in which an intervention is organized (Asakura, 2016), but also of the possibly contrasting beliefs of the intersecting cultures in which children’s lives are embedded. Indeed, measured outcomes may reflect the idiosyncratic nature of the local culture as well as behaviors valued in this specific context. On the other hand, findings may assess a more homogenized global culture centered on common human experience (Ungar, 2011).

Appealing to the children’s sociocultural background, not only facilitates them to engage in musical interactions through an expressive commonality, but also intensifies the bond between the children (Leman, 2016).

A second way of connecting to the children concerns the stance toward the children when jointly engaging in a learning process that aims at fostering resilience. Rather than developing a learning method “for” the children, musical experiences can be developed “with” the children and their environment, by jointly engaging in a spiral artistic process. In this way, the boundaries between participants and educators become fluid and flexible, where a new collective space of creativity can emerge (Schiavio and van der Schyff, 2018). Within such space, the individual aspirations, needs and interests of children merge (Abrahamson and Sánchez-García, 2016). The idea is to let children (re)discover their musical routes and (re)connect to their roots through exploring their communities’ expressive commonality using, for example, traditional stories, songs or dances that are interpreted through their twenty-first century eyes. The latter allows the individual expression, possibly differing from the common expression within the group. Children are great innovators and their voice can certainly gain more attention.

### Explore and Experiment

A second guiding principle concerns the *exploration* of individual expression through experimentation with expressive alignment of music and movement. Once children are enabled to connect to embodied experiences, they can be invited to go beyond the comfort zone of the familiar, to dive into the unknown and to engage in an environment that encourages change. Scaffolding children in movement-based musical experiences through the manipulation of environmental, task, and individual constraints, may challenge and guide them in finding their own solutions to problems or to achieve envisaged results (Bremmer and Nijs, 2020). As such, they are stimulated to shape their own experiences, through self-organization, exploration and problem-solving (Chow, 2013). This aligns with the idea of non-linear learning, which views learning as systemic, emergent, non-linear, distributed, and self-adaptive. It also aligns with the concept of informal learning, characterized by, for example, learner-initiated choices of music and collaborative work in groups and project-based learning (Green, 2009). It contrasts with traditional approaches to music and dance education, which are often based on the idea of transferring knowledge from the teacher to the student. In such an approach, learning rather occurs within structured boundaries and focuses more on the content than the context. The “instructional” nature of traditional approaches may disempower children since they involve activities that do not always align with the children’s aspirations. Therefore, an innovative joint approach should provoke children to loosen their expression, creating a non-judgmental environment in which there is plenty of room for exploration and chances to discover every child’s most authentic self. Freedom in music making and exploring with expressive alignment can reinforce new nuances in a child’s perception of what music is (e.g., Fortuna and Nijs, 2020). The same goes for movement. Moving spontaneously and authentically can question the definition of dance, and at the same time enhance feelings of body ownership and accomplishment (Bujorbarua, 2020).

### Blend

A third guiding principle concerns *blending*, whereby traditional boundaries between for example genres (western canon vs. world music) or activities (respond vs. create, performing vs. composing) are continuously crossed or even blurred to enrich children’s musical experiences and shape their musical sense-making. The basis for this principle is the idea that music is an emergent phenomenon rather than something pregiven or fixed. This resonates with Schiavio and De Jaegher (2017), who conceive the musical object as “an ongoing “open” structure that shapes and is shaped by the sense-makers in a “circular” fashion.” Considering music in this way supports combining “a love-of-process with a love-of-product” (Allsup, 2007), whereby cultural artifacts (product) such as a dance or music, maybe a starting point to further explore and experiment with (process) in order to create something new. Improvisation, i.e., a real time unpredicted event based on diverse creative skills (Biasutti, 2017), in both music and movement can become a core component of this dynamic approach for the creation of new authentic material. For example, possibilities such as voice improvisation, the use of the voice as an extension of movement and sound production involving the body (i.e., body percussion, creative storytelling, sound-painting gestures, circle songs synchronized in rhythm) can be great tools. Other ideas encompassing the exploration-experimenting mentality, could be the creation of graphic scores with body movement using paint, the use of instruments in a non-traditional way extending to the body, the use of technology for recording and producing (i.e., sampling applications on Ipad) and the connection of nature with music creation, such as soundwalks to explore the environment (Biffio, 2017). Collective composing can be used in a later stage in order to gather interesting material that has been previously created to be brought together into a new composition. During this process children learn to make independent choices and select or disregard material that has been created, prioritizing quality over effort and organizing space and time (Veloso, 2017). They also learn to accept that an ending result cannot represent the valuable procedure of creation.

The above activities demand creative and critical thinking. Having the opportunity to choose and determine certain elements of the creative process can give vulnerable children a feeling of agency and sense of purposefulness within a group. Moreover, a feeling of trust can be built between the educator/facilitator where children appreciate the fact that both sides have a common goal—to make music, move together, and create something new. For example, as involvement becomes ownership of the activity, a major shift can occur in the dynamic between the educator and the group, as well as within and between the participants (Safa, 2018).

### Cooperate

A fourth guiding principle concerns *cooperation*, through which children engage in a process of working together toward a common goal. Through co-creation of music and/or movement based on collaborative enactment, the body and body movement as mediators in the process of musical meaning formation facilitates and stimulates intersubjective interaction and participatory sense-making (Schiavio and De Jaegher, 2017). Here, the bodily dimension of human communication is intentionally addressed, and basic mechanisms of musicality are activated (Malloch and Trevarthen, 2009), by turning the body into the medium for conveying musical meaning to one’s peers (Leman, 2007). While exploration fosters autonomous musical meaning formation, cooperating embeds the individual sense-making into a process of enacting musical meaning through joint expressive alignment whereby sense-making is directly affected by the coordination of movements in an embodied interaction with the music (De Jaegher and Di Paolo, 2007). Participating in a process of joint enactment fosters the negotiation of emotional, expressive, sensorimotor, and communicative musical abilities and can therefore transform each other’s sense-making and help to create a shared world of meaning (Schiavio and De Jaegher, 2017).

Moreover, in the cooperation, the I-agency of exploration and experimentation becomes a we-agency, and individual achievement becomes a realization of the group. Children cultivate shared goals and joint problem-solving (Gaunt and Westerlund, 2013). This social interaction involved in cooperation promotes peer learning, inviting individuals to share and elaborate on different perspectives and extend their thinking beyond individual knowledge, skills, and experience (Isohätälä et al., 2017; Nielsen et al., 2018), as this helps in negotiating difference, fostering collaboration, and stimulating trust and shared forms of social understanding (Schiavio et al., 2019).

## Share

A final guiding principle we would like to propose is *sharing* one’s experiences, gathered through creative and movement-based musical activities. Sharing is a prosocial act that can take on many forms, such as attention sharing, emotion sharing, information sharing, and resource sharing (Brownell et al., 2013). Engaging in joint movement-based musical activities introduces a focus on shared decision-making, inviting the children to respond in a participatory way and thus promotes different forms of sharing. Such sharing can be seen at two levels. A first level concerns the sharing between the participating children. Through expressive alignment to music, children share a focus on the same content. At the same time, they are enabled to share the meaning they retrieve from the music through the enactment process and the articulation of their experience. Here, movements aligned to the music express the individual experience from one child to another, establishing a “me-to-you” relationship (Leman, 2007). In this way, the experience of the music becomes a shared expression, whereby children develop shared intentionality through the negotiation and renegotiations of interpretative alignments to the music. As such, children get to know each other’s perspective, develop mutual understanding, and develop communicative abilities, which are fundamental for children’s learning and creativity (Frønes, 1995). According to Kirschner and Tomasello (2010, p. 362), such shared intentionality allows “of experience each other as co-active, similar, and cooperative members of a group.” Note that children’s expressive alignments to music may be based on the culturally determined expressive commonalities or on the individual expression that emerges from explorations and experimentation.

A second level of sharing concerns sharing one’s experience with the broader community, such as family, friends, peers, teachers or care-takers. Children gain meaning and importance in what they do through presenting their work to the outer world. Showing excerpts of their own work can create a sense of reward for themselves, provoke pride and a positive sense of identity (Reynolds and Duff, 2016), but also radiate back to their family that can feel “proud” about their child’s achievement. Moreover, after creating and presenting, the valuable process of evaluation follows, where they can discover what worked and what did not work so well. In this way children can progress into an ongoing process of personal growth and empowerment.

A viable way to share this ongoing process is the use of pedagogical documentation, an enhanced form of observation that gives children a voice by creating “an environment that documents not only the results but also the processes of learning and knowledge building, which narrate the didactic paths and state the values of reference” (Ceppi and Zini, 1998, p. 25). Based on, for example, drawings, photos, videos, transcriptions of children’s talking, and diaries, it generates a visible trace of the children’s experiences and learning trajectories. This enables sharing and (jointly) revisiting experiences. On the one hand it promotes the emergence, deepening, and strengthening of understandings, allowing children to learn from each other and to be stimulated by others’ work (Buldu, 2010). On the other hand, pedagogical documentation is an ideal medium to reach out to the community. It allows ongoing interpretation involving teachers, children, families and the wider community (Merewether, 2018).

An interesting aspect of pedagogical documentation, especially in the context addressed in this paper, is its desire to understand children’s abilities without any predetermined normative framework (Dahlberg et al., 2006). In this sense, it helps adults see and understand children as individuals rather than normalizing them against standardized measures and categorizing some “normal” and some “abnormal” (Moss et al., 2000), or for that matter, “talented” or “untalented,” “easy” or “difficult.” Rather, it puts the child at the centre, avoiding an adult perspective that disconnects from the child’s experience (Eckhof, 2019).

## Conclusion

In this paper, we discursively elaborated on the potential of combining music and movement to support building resilience, which is a necessary condition for refugee children, and by extension to all children at risk, to have a flourishing life. The main idea of this work is that, due to the tight connection between music and movement and the empowering nature of this connection, engaging children in movement-based musical activities can make a viable contribution to the development of resilience.

However, such activities need careful consideration, especially when working with vulnerable children such as refugee children. Therefore, we formulated a set of guiding principles for the design designing of movement-based musical activities. In our view, the elaborated theoretical background and the associated guiding principles may contribute to both research and practice projects that investigate and promote the social impact of music, working for example with refugee children. The contribution of this work lies in the emphasis on the importance of addressing the bodily basis of musical engagement to promote the development of resilience. Indeed, despite the above elaborated connections between music, movement, eudaimonia and resilience, it appears that in the domain of socio-artistic musical practices, the full potential of this connection has not yet been addressed. Often, projects with refugee children are about music making or about dancing, but seldom about how both can be combined. Based on the presented work, we believe that activities combining both music and movement in the creative process could significantly enhance the potential of these interventions in promoting resilience, by addressing both internal and external protective factors and, as such, by developing the key components of resilience.

The proposed theoretical and pedagogical framework may have important implications for future research, practice and policy. Regarding research, both applied and fundamental research can depart from the ideas we presented. The former may address the development of good practices in relation to different contexts (e.g., refugees or orphans) and to the different presented guiding principles. While the presented framework may contribute to operationalizing the concept of resilience, the developed good practices may become the basis for intervention-based studies. The latter may address the processes that underlie optimal interactions (e.g., coordination, optimal experience) and their effect on resilience, operationalized based on its internal (e.g., agency and self-regulation, self-esteem) and external factors (e.g., prosocial behavior).

Regarding practice, teachers and teacher trainers may be inspired to broaden their practices by systematizing the use of body movements, embedding the learning goals in the proposed framework, and by shaping their learning activities based on the proposed principles. Indeed, there is a growing interest in socio-artistic practices, as reflected in current changes in the curriculum of higher music education institutions, where the training of future teachers is no longer solely targeted on formal educational contexts. This is an important step, taking into account the growing number of children that seek refuge or migrate to European and society’s responsibility to support these children in their pursuit of a flourishing life. As we have seen, music and movement may contribute significantly to this by helping them to build resilience. However, the training of socio-artistically oriented educators is still in its infancy. Therefore, our work may contribute to the design of teacher training curricula that prepare future teachers who want to want to work with refugee or other vulnerable children.

Regarding policy, the proposed frameworks may convince leaders in our society about the importance of engaging children in empowering musical experiences, not only to promote the personal and artistic growth of the individual, but also to strengthen society. Indeed, supporting refugee children to build resilience and develop a flourishing live, contributes to building and maintaining inclusive and diverse communities and societies. Our work contributes to informing policy makers about this potential and provides a framework that may help them to implement research-based findings in decisions on, for example, curricula and funding.

Finally, we believe that the proposed framework may also be of benefit to the development of formal music education and contribute to the personal growth of all children and adults. In times where music education is challenged due to societal changes, funding issues and the undervaluing of the arts in society, music education is in need of new frontiers. Indeed, the call for inclusion and diversity is paramount, in our society in general but also in music education in particular. Integrating findings as presented here, may help to shape a music education of the future, in which children of all cultural or socio-economical backgrounds may find a place to develop into creative and resilient, open-minded and sociable individuals and to acquire enriching experiences, knowledge and skills that contribute to a flourishing life.

## Author Contributions

Both authors have contributed equally to writing the manuscript.

## Conflict of Interest

The authors declare that the research was conducted in the absence of any commercial or financial relationships that could be construed as a potential conflict of interest.

## Footnotes

1 https://righttoplay.com/en/
